# Impact of Dietary Carbohydrate/Protein Ratio on Hepatic Metabolism in Land-Locked Atlantic Salmon (*Salmo salar* L.)

**DOI:** 10.3389/fphys.2018.01751

**Published:** 2018-12-06

**Authors:** Mónica B. Betancor, Rolf E. Olsen, Lucie Marandel, Ole F. Skulstad, Angelico Madaro, Douglas R. Tocher, Stephane Panserat

**Affiliations:** ^1^Institute of Aquaculture, Faculty of Natural Sciences, University of Stirling, Stirling, United Kingdom; ^2^Department of Biology, Norwegian University of Science and Technology, Trondheim, Norway; ^3^INRA-UPPA, UMR 1419, Nutrition Metabolism and Aquaculture, Aquapôle, Institut National de la Recherche Agronomique, Paris, France; ^4^Norwegian Institute of Marine Research, Bergen, Norway

**Keywords:** salmon populations, dietary carbohydrates, transcriptomics, glucose metabolism, land-locked

## Abstract

A common-garden experiment was carried out to compare two genetically distinct strains of Atlantic salmon (*Salmo salar*) fed diets with either high (CHO) or low (NoCHO) digestible carbohydrate (starch). Twenty salmon from either a commercial farmed strain (F) or a land-locked population (G) were placed in two tanks (10 fish of each population in each tank) and fed either CHO or NoCHO feeds. At the end of the experiment fish were fasted for 8 h, euthanized and blood and liver collected. Both diet and population had an effect on circulating glucose levels with G showing hypoglycaemia and dietary starch increasing this parameter. In contrast, G showed increased plasma triacylglycerol levels regardless of dietary treatment suggesting faster conversion of glucose to triacylglycerol. This different ability to metabolize dietary starch among strains was also reflected at a molecular (gene) level as most of the metabolic pathways evaluated were mainly affected by the factor population rather than by diet. The data are promising and suggest different regulatory capacities toward starch utilization between land-locked salmon and the farmed stock. Further analyses are necessary in order to fully characterize the capacity of land-locked salmon to utilize dietary carbohydrate.

## Introduction

Aquafeeds have traditionally depended on the use of fishmeal derived from marine fisheries as the main protein source (Tocher, 2003). However, aquaculture is the fastest growing animal protein producing sector and, in order to supply the global demand for fish, marine-derived dietary ingredients should be replaced with plant-based ingredients that can be naturally rich in carbohydrates/starch (Panserat and Kaushik, 2010). The ability of fish to use dietary starch varies greatly among species, mainly in relation to their feeding habits (Polakof et al., 2012; Kamalam et al., 2017). In this sense, strictly carnivorous teleost species, such as Atlantic salmon (*Salmo salar*), are metabolically adapted for high catabolism of proteins and low utilization of dietary starch (Hemre et al., 2002). These species are considered to be “glucose-intolerant" as reflected by the persistent hyperglycaemia after intake of starch-enriched meals (reviewed in Polakof et al., 2012). Given that most of the key genes involved in pathways of carbohydrate metabolism are present in fish, the poor utilization of dietary starch in carnivorous fish compared to herbivorous teleosts/mammals may be due to atypical hormonal and nutritional regulation as a result of evolutionary adaptation (Enes et al., 2009; Polakof et al., 2011a). However, previous studies have also demonstrated that selected lines of rainbow trout (*Oncorhynchus mykiss*) showed increased capacity to metabolize dietary starch (Skiba-Cassy et al., 2009; Kamalam et al., 2012), linking enhanced *de novo* lipogenesis to better glucose homeostasis (Panserat et al., 2009; Polakof et al., 2011b).

Land-locked salmon populations, found in several freshwater bodies in both Europe and North America, are characterized by completing their whole life-cycle in lakes and rivers instead of migrating to the ocean. This may suggest that land-locked salmon might be subjected to higher dietary contents of carbohydrates compared to their anadromous counterparts that feed on prey rich in protein and lipid in the open sea, as has been shown recently with brown trout (*Salmo trutta*) in Kerguelen island (Marandel et al., 2018). By analyzing the gut contents of resident Kerguelen trout, the authors concluded that fish mainly fed on terrestrial invertebrates from the phylum Arthropoda that were relatively rich in glycogen (Marandel et al., 2018). Land-locked salmon are an interesting and valuable genetic resource as certain traits associated with life in ancestral anadromous populations (such as better utilization of protein/lipids as an energy source) may have been lost/suppressed, and other traits such as utilization of dietary carbohydrate may be enhanced due to the different selection pressures (Nilsen et al., 2008). In this respect, a previous trial suggested that a land-locked Atlantic salmon population (Gullspång Swedish stock) had higher lipogenic and glycolytic potential than a farmed strain, as well as decreased lipolysis when fish were fed differing contents of omega-3 long-chain polyunsaturated fatty acids (n-3 LC-PUFA) (Betancor et al., 2016). These results appeared to indicate an increased potential to utilize dietary carbohydrate/starch as a source of energy together with lipid sparing in liver of land-locked salmon. It was hypothesized that intermediary metabolism may differ among the two populations and this might be related to feeding habit as anadromous salmon encounter essentially no carbohydrate in their natural diet and thus could have lost some of their metabolic capacity to utilize significant amounts of dietary carbohydrate. In this context, there is no information regarding the impact that high carbohydrate feeds could have in a population of land-locked Atlantic salmon.

In order to elucidate the metabolic potential to utilize dietary carbohydrate (starch) of a land-locked salmon population, a common-garden experiment was performed. Common-garden experiments involve the comparison of genetically distinct strains, families or populations under identical environmental conditions, such experimental protocols being often used to differentiate the effects of genetic and environmental variation on phenotype. To achieve this, two feeds containing high (CHO) or low (NoCHO) levels of digestible dietary starch at the expense of protein (42% vs. 65%) were tested over 32 days in two Atlantic salmon populations, the land-locked Swedish Gullspång stock, and the Norwegian national farmed stock, Aquagen strain. Effects of feeding high levels of starch were determined on plasma biochemistry, hepatic transcriptomic response and candidate gene expression (qPCR) in liver.

## Materials and Methods

### Ethics Statement

The experiment was carried out in accordance with Norwegian national legislation via the Norwegian Animal Welfare Act (LOV-2015-06-09-19-65) Regulations on the Use of Animals in Experiments (FOR-2017-04-05-451) that was amended to implement the requirements contained in the European (EU) Regulations for the use of animals in scientific experimentation (Directive 2010/63/EU). Norway fully implemented the EU Directive within its legislature on 1 August 2016 via the European Economic Area Agreement. In addition, the experiment was separately assessed and approved by the Animal Welfare and Ethical Review Board of the University of Stirling (Approval AWERB 16-17 143).

### Experimental Feeds

Two isoenergetic feeds based on fishmeal and fish oil as the main protein and lipid sources, respectively, were manufactured as extruded pellets by INRA (Donzacq, Landes, France). Feeds were formulated to satisfy the nutritional requirements of salmonid fish (National Research Council [NRC], 2011) and contained either high (CHO) or low (NoCHO) contents of carbohydrate using gelatinized corn starch as the digestible carbohydrate source (Table 1). Fishmeal was used as the protein source given that high dietary carbohydrate levels can lead to palatability issues in salmonids and, therefore, reduced feed intake (Kamalam et al., 2017). Thus, the two diets were formulated with carbohydrate/protein ratio as the main factor with the high starch CHO diet containing a lower level of dietary protein compared to the NoCHO diet. The high level of dietary starch investigated meant it was not practical to replace carbohydrate in the NoCHO diet by cellulose to maintain the same level of protein.

**Table 1:** Formulation and proximate composition of the two experimental feeds.

	NoCHO	CHO
*Ingredients (%)*		
Fish meal^1^	90.85	55.90
Fish oil^1^	5.15	10.10
Starch^2^	-	30.00
Vitamin mix^3^	1.00	1.00
Mineral mix^4^	1.00	1.00
Alginate^5^	2.00	2.00
*Proximate composition*		
DM (%Feed)	95.38	93.19
Crude protein (% DM)	65.25	41.80
Crude lipid (% DM)	14.99	12.21
Gross energy (kJ g^−1^ DM)	22.92	22.80
Ash (% DM)	15.62	10.72
Carbohydrates (% DM)	<1.00	27.28

*DM, dry matter. ^1^Sopropeche, Boulogne-sur-Mer, France. ^2^Gelatinized corn starch (Roquette, Lestrem, France). ^3^Supplying (kg^−1^ diet): DL-α-tocopherol acetate 60 IU, sodium menadione bisulphate 5 mg, retinyl acetate 15000 IU, DLcholecalciferol 3000 IU, thiamin 15 mg, riboflavin 30 mg, pyridoxine 15 mg, vit. B12 0.05 mg, nicotinic acid 175 mg, folic acid 500 mg, inositol 1000 mg, biotin 2.5 mg, calcium panthotenate 50 mg, and choline chloride 2000 mg. ^4^Supplying (kg^−1^ diet): calcium carbonate (40% Ca) 2.15 g, magnesium oxide (60% Mg) 1.24 g, ferric citrate 0.2 g, potassium iodide (75% I) 0.4 mg, zinc sulfate (36% Zn) 0.4 g, copper sulfate (25% Cu) 0.3 g, manganese sulfate (33% Mn) 0.3 g, dibasic calcium phosphate (20% Ca, 18% P) 5 g, cobalt sulfate 2 mg, sodium selenite (30% Se) 3 mg, potassium chloride 0.9 g, and sodium chloride 0.4 g. ^5^Louis François, Marne-la-Vallée, France.*

### Fish and Dietary Trial

Two different Atlantic salmon populations were used in this trial. Eggs from a land-locked Atlantic salmon population (Lake Vanern/Gullspång Swedish stock; Termed G) and a Norwegian farmed stock (Termed F, Aquagen strain) were hatched at IMR, Matre. The following year, the fish underwent smoltification in May (1+) using natural light regime and were transferred into seawater. There were no mortalities following seawater transfer and fish were maintained on standard commercial diets until start of the experiment. A common – garden experiment was set up in which 40 fish (approx. 3000 g weight; 20 fish per population) were individually PIT-tagged and placed into one of two experimental seawater tanks, each one containing 10 fish from each population. One of the tanks was fed with diet CHO, while the other was fed with diet NoCHO over a period of 32 days at a water temperature of 8°C and natural photoperiod. At the end of the feeding trial fish were fasted for 8 h, euthanized with metacaine sulfonate (MS-222; Finquel Argent Chemical Laboratories, Redmond, WA, United States) and blood from each fish was collected via the caudal vein by heparinized vacutainers and centrifuged to obtain plasma, which was stored at −70°C until further analysis. In addition, samples of liver were quickly collected, stabilized in RNA Later (Sigma, Poole, United Kingdom) according to the manufacturer’s protocol and stored at −20°C prior to RNA extraction. Tanks were supplied by seawater using a flow-through system. Oxygen (Oxyguard 420 probe, Oxyguard International, Denmark), temperature (TST 487-1A2B temperature probes) and salinity (Liquisys MCLM223/253 probes) were measured continuously and recorded at tank level, with no differences recorded between tanks.

### Chemical Analysis of Diets

The chemical composition of diets were analyzed by the following procedures: protein content (*N* = 6.25) was determined by Kjeldahl method after acid digestion; total lipid was determined by automated petroleum ether extraction (Soxtherm, C. Gerhardt GmbH & Co., Königswinter, Germany), gross energy was determined in an adiabatic bomb calorimeter (IKA, Heitersheim Gribheimer, Germany); starch content was determined by a kit using an enzymatic method (Invivo Labs, France).

### Plasma Metabolites

Plasma glucose levels were determined using a commercial kit (Biomérieux, Marcy l’Etoile, France) adapted to a microplate format. Plasma triglyceride levels were measured by a colorimetric enzyme assay using hepatic lipase (EC 3.1.1.3), glycerokinase (EC 2.7.1.30), glycerol-3-phosphate oxidase (EC 1.1.3.21), and peroxidase (EC 1.1.11) (PAP 150 kit; Biomérieux, Marcy-l’étoile, France).

### RNA Extraction

Liver from all 10 individual fish per dietary treatment and population (*n* = 40) were homogenized in 1 ml of TriReagent^®^ (Sigma-Aldrich, Dorset, United Kingdom) RNA extraction buffer using a bead tissue disruptor (Bio Spec, Bartlesville, Oklahoma, United States). Total RNA was isolated following manufacturer’s instructions, purified using a commercial kit (RNeasy Mini Kit, Qiagen, Manchester, United Kingdom) and quantity and quality determined by spectrophotometry using a Nanodrop ND-1000 (Labtech Int., East Sussex, United Kingdom) and electrophoresis using 500 ng of total RNA in a 1% agarose gel. To perform the RNA amplifications, 2500 ng RNA from each of two fish from the same tank and population were pooled to obtain five samples per treatment and stock (*n* = 5).

### Liver Transcriptome – Microarray Hybridizations and Image Analysis

Transcriptomic analysis was performed in liver using a custom-designed 4 × 44 k Atlantic salmon oligo microarray (Agilent Technologies, Wokingham, United Kingdom; ArrayExpress accession no. A-MEXP-2065). The salmon microarray and laboratory procedures utilized in this study have been widely used and validated in many previous studies (Morais et al., 2012; Tacchi et al., 2012; Bicskei et al., 2014; Martinez-Rubio et al., 2014; Betancor et al., 2015a,b). Replicate RNA samples were amplified using TargetAmp^TM^ 1-Round Aminoallyl-aRNA Amplification Kit, (Epicentre Technologies Corporation, Madison, Wisconsin, United States) following recommended procedures. Aminoallyl-amplified RNA (aRNA) samples were labeled with Cy3 dye (GE HealthCare Life Sciences, Buckinghamshire, United Kingdom) while a pool of all aRNA samples was labeled with Cy5 dye (GE HealthCare Life Sciences) and was used as a common reference in a dual-label common reference design and finally hybridized. Scanning was performed using a GenePix 4200 AL Scanner (Molecular Devices (United Kingdom) Ltd., Wokingham, United Kingdom), and the resulting images analyzed with Agilent Feature Extraction Software v.9.5 (Agilent Technologies) to extract the intensity values and identify the features. Features considered outliers (i.e., defined as those probes whose background intensity was between the 0.05 and 99.95th percentile of the distribution) in two or more replicates within at least one treatment were excluded from further analyses. Additionally, features consistently expressed just above background noise (defined as those features whose intensity was lower than 5th percentile of the distribution in 75% or more of the analyzed samples) were also removed. The full protocol for microarray laboratory and data analysis has been reported previously (Betancor et al., 2015a). The full data set supporting the results is available in MIAME-compliant format in the ArrayExpress repository under accession number E-MTAB-6188.

### Gene Ontology Analysis

Gene ontology (GO) analysis was performed using the functional annotation cluster from the DAVID (Database for Annotation, Visualization and Integrated Discovery) bioinformatics resource (da Huang et al., 2009a,b). The IDs of genes exclusively affected by population were used as input and processed with the functional annotation cluster from DAVID. The resulting data were used as input in the Enrichment Map App from the Bader Lab (Merico et al., 2010, 2011) in the Cytoscape network visualization tool (version 3.5.1) (Cline et al., 2007). Selected mRNA probes differentially expressed by population and assigned to be part of significantly affected GO process were used as an input in ClueGo (Bindea et al., 2009, 2013) which visualizes the selected terms in a functionally grouped annotation network that reflects the relationships between the terms based on the similarity of their associated genes.

### Quantitative PCR Gene Expression Analysis in Liver

Gene expression levels were determined by real-time quantitative RT-PCR in liver as previously described (Betancor et al., 2015b; Marandel et al., 2016; Panserat et al., 2017). The expression of the following genes was examined: *g6pca*, *g6pcb1*, *fbp1a*, *fbp1b1*, *fbp1b*, and *pck2* for gluconeogenesis, glucose transporter 2 (*glut2*), *gckb*, and *pkl* for glucose transport and glycolysis, *acly*, *fas*, and glucose-6-phosphate dehydrogenase (*g6pdh*) for lipogenesis and biosynthesis of pentose phosphates, carnitine palmitoyltransferase 1 isoforms A and B (*cpt1a* and *cpt1b*) and 3-hydroxyacyl-CoA dehydrogenase (*hoad*) for fatty acid β-oxidation and *qcr2*, *atp5a*, *cox4*, *shdb*, and *cs*, for mitochondrial metabolism (Supplementary Table 1). The primers were designed from rainbow trout sequences (Marandel et al., 2016; Panserat et al., 2017); all the RT-PCR products were thus sequenced to check their identity in Atlantic salmon.

### Statistical Analysis

Microarray hybridization data were analyzed in GeneSpring GX version 12.6.1 (Agilent Technologies, Wokingham, Berkshire, United Kingdom) by two-way analysis of variance (ANOVA), which examined the explanatory power of the variables, diet and population, as well as “diet x population” interaction. No multiple test correction was employed as previous analyses indicated that they were over-conservative for these nutritional data (Morais et al., 2011). Data were submitted to the Kyoto Encyclopedia of Genes and Genomes (KEGG) (Kanehisa and Goto, 2000) for biological function analysis.

The results of plasma metabolites and gene expression are presented as means ±*SD* The effects of diets, populations and the diet population interaction on the different parameters were tested using R software (v3.1.0)/R Commander by means of a two-way ANOVA with diets and populations as independent variables. Before statistical analysis, normality was assessed using the Shapiro-Wilk test, while homeoscedasticity was determined using Levene’s test. Differences were considered statistically significant at *P* < 0.05. When interactions were significant, means were compared using one-way ANOVA (*P* < 0.05).

## Results

### Plasma Metabolites

**FIGURE 1:**
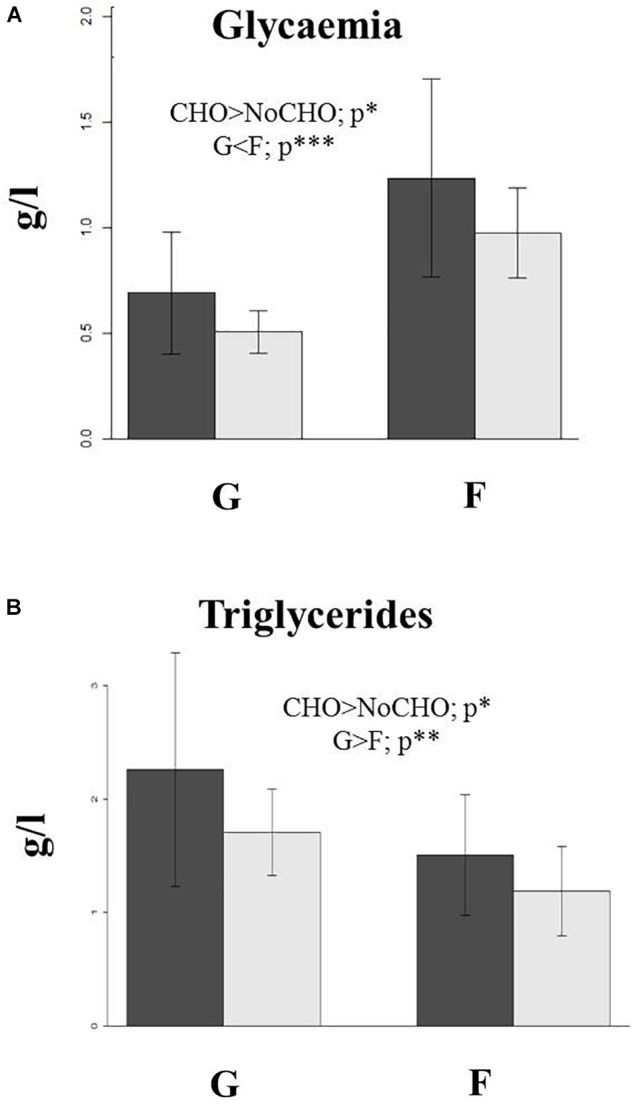
**(A)** Plasma glucose and **(B)** triglyceride levels in land-locked (G) and farmed (F) Atlantic salmon after being fed with a diet high (dark bars) or low (light bars) in carbohydrate for 32 days. ^∗^*p* < 0.05; ^∗∗^*p* < 0.05; and ^∗∗∗^*p* < 0.001.

Data on post-prandrial plasma glucose level showed the effect of diet (*p* = 0.034), with salmon fed CHO showing hyperglycaemia 8 h after feed intake (Figure 1A). Plasma glucose levels were also affected by strain as land-locked salmon had significantly lower circulating glucose than the farmed stock (*p* < 0.0001). Significant differences in plasma triglyceride were also found among fish fed the two dietary treatments (*p* = 0.044) with fish fed CHO showing higher levels (Figure 1B). The factor “population” also affected triglyceride levels in plasma (*p* = 0.004) with land-locked salmon showing higher values.

### Liver Transcriptome Response

**FIGURE 2:**
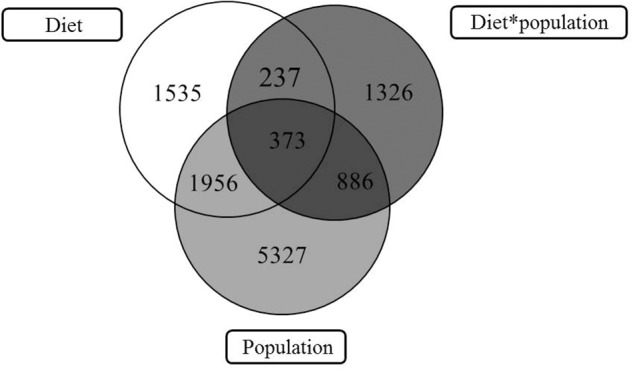
Venn diagram representing mRNA transcripts in liver differentially expressed in response to diet, population, and those showing interaction between diet and population.

**FIGURE 3:**
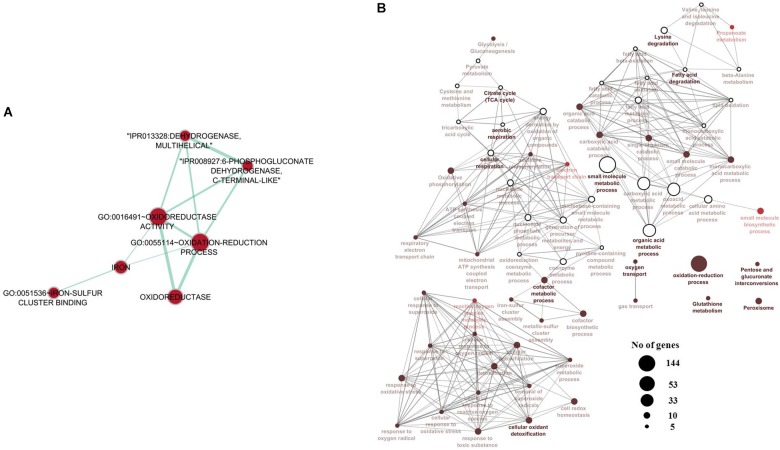
**(A)** Pathway clustering analysis performed on the 5327 genes exclusively affected by population. **(B)** Enriched functions and pathways of the metabolism-related genes exclusively related to population. The network of pathways was created with ClueGo and CluePedia Cytoscape apps. The pathways were functionally grouped and interconnected based on the kappa score. The size of the nodes indicates the number of genes associated. Pathways are colored based on their significance after Bonferroni correction, where the most significant terms are shown in dark red and the least significant terms are illustrated in white.

**FIGURE 4:**
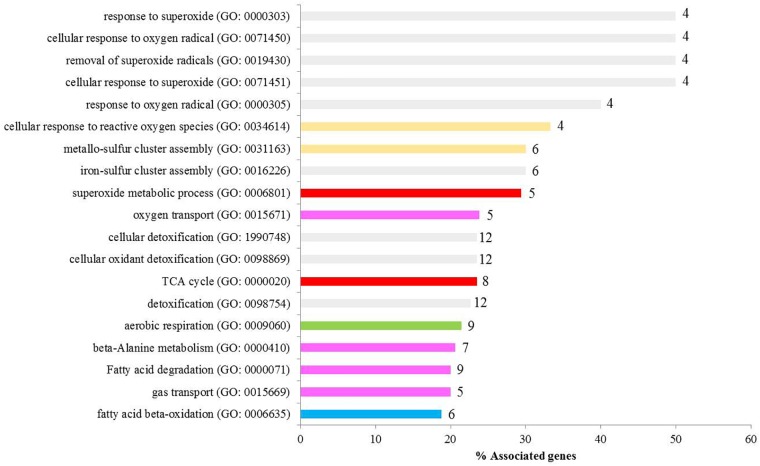
GO analysis of differentially expressed metabolism-related genes indicating the percentage of genes related (X axis) as well as the number of genes affected. Gray bar, cellular oxidation detoxification; yellow bar, oxygen transport; red bar, cofactor metabolic process; pink bar; citrate cycle; green bar, cellular respiration; and blue bar, organic acid metabolism.

Two-way ANOVA showed that a higher number of genes/probes were affected by “population” than “diet,” with the number of probes affected by the interaction of factors being much lower (Table 2). Additionally a higher number of probes were affected at the lowest *p*-value by the factor “population” than “diet” (Table 2). In total 8542 probes were affected by the factor population, with 5327 being exclusively affected by the factor population but not the diet factor (Figure 2). Likewise, 4101 probes were shown to be differentially regulated by diet, with 1535 probes affected only by diet, and a further subset of 1326 probes only affected by the interaction of both factors, population and diet (Figure 2). The list of probes exclusively affected by population (5327) was submitted to DAVID and an enrichment map built showing a series of interconnected GO categories in order to elucidate which pathways were affected by the genetic background of the fish, with one of the clusters belonging to metabolic processes (Figure 3A; 2724 individual annotated genes). Analyzing this cluster of genes with Cluego showed the differentially regulated genes were mainly associated with small molecule metabolic processes such as citrate cycle, lysine and fatty acid degradation or organic acid metabolic process (Figure 3B). Another category highly enriched was that of cellular oxidation and detoxification (Figure 3B). When restricting to the top 20 terms according to the percentage of genes found compared to all genes associated with the term, many of the terms were related to the category “cellular oxidation detoxification” followed by “oxygen transport,” “cofactor metabolic process,” “citrate cycle,” “cellular respiration” and “organic acid metabolism” (Figure 4).

**Table 2:** Summary of the results of the two-way ANOVA indicating number of probes differentially expressed and *p*-values (FC > 1.3).

	*P* < 0.05	*P* < 0.02	*P* < 0.01	*P* < 0.005	*P* < 0.001
*p*-value (D)	4101	2305	1434	877	270
*p*-value (D^∗^P)	2822	1370	808	459	107
*p*-value (P)	8542	6541	5393	4403	2606

*D, diet; P, population.*

**FIGURE 5:**
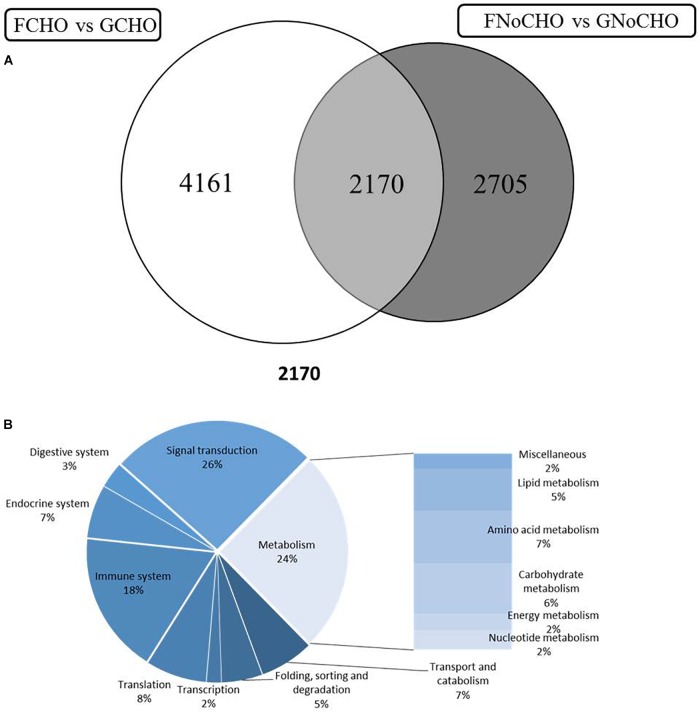
**(A)** Venn diagram representing mRNA transcripts differentially expressed in liver of farmed (F) or land-locked (G) Atlantic salmon fed either high (CHO) or low (NoCHO) carbohydrate feeds. **(B)** Distribution by category of common differentially expressed genes (2170) in liver of Atlantic salmon fed high or low CHO feeds.

Comparing fish from the two populations fed the same diet, a higher number of genes were differentially expressed when comparing fish fed high carbohydrate (diet CHO; 6331 DEG, *p* < 0.05; FC > 1.3) compared with fish fed low carbohydrate (diet NoCHO; 4875 DEG), with 2170 genes commonly regulated (Figure 5A). When submitted to KEGG, the genes commonly affected between both contrasts (2170) showed high representation of “signal transduction” (26%) followed by “metabolism” (24%) and “immune system” (18%) (Figure 5B). Within “metabolism,” carbohydrate metabolism was the second most represented category (6%) following amino acid metabolism (7%). When the list of DEG was restricted to the top 100 probes according to fold-change (FC), an increased proportion of “metabolism” was evident (51.1%), with lipid and carbohydrate metabolism being the two categories mainly affected (Supplementary Table 2). It was also noteworthy that all genes followed the same direction (up- or down-regulated) in both comparisons although, in general terms, the FC was higher when comparing the two populations of fish fed diet CHO. Genes that were up-regulated in farmed fish were related to lipid metabolism, with one gene, *elongation of very long chain fatty acids protein 6*, represented by two features at very high FC (approximately 11) (Supplementary Table 1). Among the genes that were down-regulated in farmed fish were catabolic genes such as *carnitine O-palmitoyltransferase 1* and *phosphatidylserine decarboxylase*. Similarly, among carbohydrate metabolism genes, a higher FC was observed among fish fed diet CHO and genes followed the same direction of regulation in both contrasts (FCHO vs. GCHO and FNoCHO vs. GNoCHO), with the highest regulation in *polypeptide N-acetylgalactosaminyltransferase* (FC = 33.7 in FCHO vs. GCHO). The gene *glyceraldehyde 3-phosphate dehydrogenase* was represented by two features, both down-regulated in farmed salmon (Supplementary Table 2).

### Candidate Gene Expression Profile in Liver

In order to have a specific view of the effects of dietary carbohydrate level on the two populations, some target genes known to code for key proteins involved in postprandial use of dietary glucose in liver were analyzed by qPCR. In general, good correspondence in terms of intensity (FC) and direction of change (up- or down-regulation) was observed among the genes studied by qPCR and the corresponding transcripts detected in the microarray (Supplementary Table 3).

### Glucose Transport and Glycolysis

**FIGURE 6:**
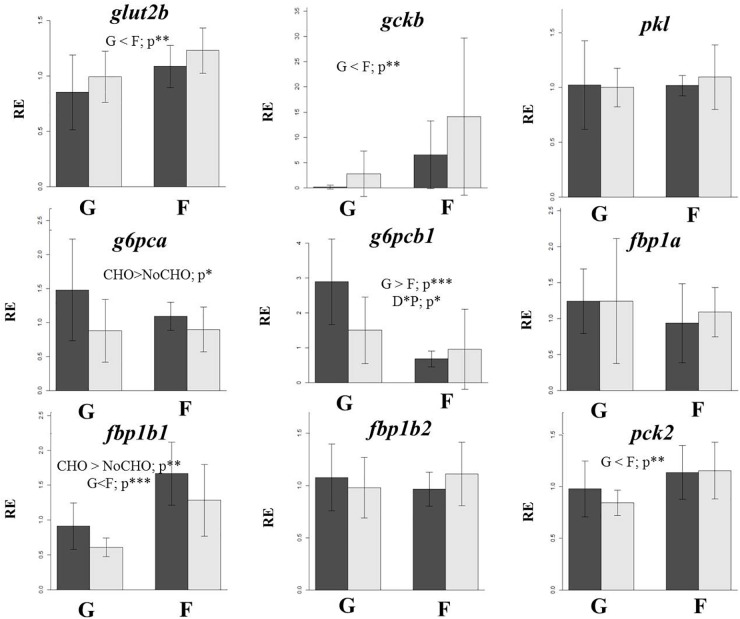
Gene expression of a glucose transporter and selected glycolytic and gluconeogenic genes in the liver of land-locked (G) or farmed (F) Atlantic salmon fed a diet high (dark bars) or low (light bars) in carbohydrate for 32 days. *glut2b*, Glucose transporter type 2; *gckb*, glucokinase paralog b; *pkl*, liver pyruvate kinase; *g6pca*, glucose 6-phosphatase paralog a; *g6pcb1*, glucose 6-phosphatase paralog b1; *fbp1a*, fructose 1,6-bisphosphatase 1 paralog a; *fbp1b1*, fructose 1,6-bisphosphatase 1 paralog b1; *fbp1b2*, fructose 1,6-bisphosphatase 1 paralog b2; *pck2*, mitochondrial phosphoenol pyruvate kinase. ^∗^*p* < 0.05; ^∗∗^*p* < 0.05; and ^∗∗∗^*p* < 0.001.

The factor population affected the expression of the glucose transporter gene *glut2b* and the glycolytic enzyme *gckb*, with lower expression in land-locked salmon compared to farmed salmon, while the expression of *pkl* was not affected by fish population. Dietary carbohydrate level did not regulate the expression of any of these three genes (Figure 6).

### Gluconeogenesis

Dietary carbohydrate level only regulated the expression of two out of the six gluconeogenic genes studied, with lower relative expression levels of *g6pca* and *fbp1b1* in fish fed NoCHO (Figure 6). The factor population also affected expression of three gluconeogenic genes although the sense of regulation varied, with *fbp1b1*and *pck2* showing lower expression and *g6pcb1* showing higher expression in land-locked salmon relative to farmed salmon. The expression of *fbp1a* and *fbp1b2* showed no variation between dietary treatments or populations.

### Mitochondrial Metabolism

**FIGURE 7:**
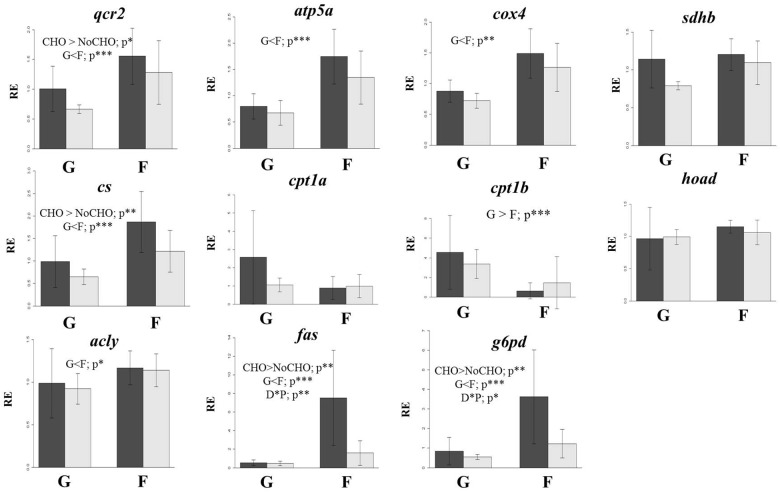
Gene expression of selected mitochondrial metabolism, β-oxidation, lipogenesis and biosynthesis of pentose phosphate genes in the liver of land-locked (G) or farmed (F) Atlantic salmon fed a diet high (dark bars) or low (light bars) in carbohydrate for 32 days. *qcr2*, ubiquitinol cytochrome c reductase core protein 2; *atp5a*, ATP synthase form 5; *cox4*, cytochrome oxidase 4; *sdhb*, succinate dehydrogenase complex iron sulfur subunit B; *cs*, citrate synthase; *cpt1a*, carnitine palmitoyl transferase isoform 1a; *cpt1b*, carnitine palmitoyl transferase isoform 1a; *hoad*, hydroxyacyl-CoA dehydrogenase; *acly*, ATP citrate lyase; *fas*, fatty acid synthase; *g6pd*, glucose 6-phosphate dehydrogenase. ^∗^*p* < 0.05; ^∗∗^*p* < 0.05; and ^∗∗∗^*p* < 0.001.

Expression levels of two of the studied genes, *qcr2* and *cs*, were altered by both dietary carbohydrate level and population, with expression levels being lower in NoCHO-fed fish, and in land-locked salmon (Figure 7). Population strongly affected the other two genes (*atp5a* and *cox4*) with lower relative expression levels in land-locked salmon.

### β-oxidation

Of the three studied genes, only *cpt1b* showed differential expression, being higher in land-locked salmon (Figure 7).

### Lipogenesis and Biosynthesis of Pentose Phosphate

The liver of landlocked salmon showed lower expression of *acly* regardless of dietary carbohydrate level (Figure 7). The expression of both *fas* and *g6pd* were influenced by dietary carbohydrate level and population, with higher expression in fish fed diet CHO and lower expression in land-locked salmon (Figure 7). In addition, interaction between dietary carbohydrate level and population was observed for these two genes.

## Discussion

In light of previous findings, the present study hypothesized that land-locked salmon had a higher capability to metabolize dietary carbohydrate (i.e., starch) than the farmed stock (Betancor et al., 2016). However, the land-locked population had not been challenged with a high carbohydrate diet as the previous study aimed to explain the potential of land-locked salmon to biosynthesize n-3 LC-PUFA. In the present study, dietary carbohydrate intake led to higher post-prandial glycaemia in both populations as expected (Hemre et al., 1996; Panserat et al., 2000; Kamalam et al., 2012). However, the glycaemia level was more moderate in land-locked fish than in farmed salmon (approximately 0.5 FC lower) regardless of diet, which indicated a better ability to manage post-prandial plasma glucose levels in land-locked salmon. Similarly, a study with two lines of rainbow trout showed lower plasma glucose levels in fish selected for greater muscle fat (Skiba-Cassy et al., 2009). However, the response was more moderate and differences were observed only 24 h after feeding, with the response not as acute as observed in the present study. This different capacity to deal with glycaemia in land-locked salmon was associated with changes at a molecular level in all of the metabolic pathways evaluated in the present study. However, further studies including a glucose tolerance test would be necessary in order to fully characterize the metabolic capacity of landlocked salmon to utilize dietary carbohydrates.

### Glucose Metabolism and Transport in Liver: Diets and Populations Effects

In this respect, land-locked salmon exhibited lower expression of the glucose transporter *glut2b*. Interestingly, high glucose was found to have no effect on *glut2b* expression level in a previous study in salmonids (Jin et al., 2014). GLUT2 is the major glucose transporter in the plasma membrane of hepatocytes in mammals and is bi-directional, taking up glucose for glycolysis and releasing glucose during gluconeogenesis (Thorens et al., 1988). Therefore, the decreased expression of this enzyme in land-locked salmon could explain, at least in part, why this population was hypoglycaemic as there was an inhibition of glucose release. However, given the bi-directionality of GLUT2, a reduction in its activity could also mean a decrease in the uptake of glucose for glycolysis. Indeed, land-locked salmon also showed lower expression of *gck*, which metabolizes glucose when glucose is in excess (Panserat et al., 2014). It is thought that *gck* expression levels in the liver are related to glycaemia levels (Postic et al., 2001), and this was observed in the present trial. In addition, in rats (a carbohydrate-dependent mammal) it is known that the translocation of *gck* from the nucleus to the cytoplasm is quite rapid (20–30 min). Therefore, it appears that land-locked salmon could better regulate glucose homeostasis as shown by the low glycaemia with low *gck* gene expression. Globally, one of the hypotheses of the present study, that land-locked salmon had a higher glycolytic potential as suggested by transcriptomic analysis previously (Betancor et al., 2016), was not confirmed in the present study by analyzing the two glycolytic actors *gck* and *pkl*.

In contrast, the mRNA levels of the gluconeogenic gene *g6pcb1* (catalyzing the last step of the gluconeogenesis, the glucose-6-phosphatase enzyme) were higher in land-locked salmon, which may suggest that carbohydrate-fed land-locked salmon showed enhanced gluconeogenesis, but this result is not consistent with the lower plasma glycaemia in this population. Nevertheless, the expression of *g6pca* (another gene coding glucose-6-phosphatase) was not affected by population, but was regulated by dietary carbohydrate with high levels leading to increased expression. It should be noted that a recent study in rainbow trout evaluating the fate of duplicated genes after the salmonid-specific whole genome duplication (Ss4R) found that *g6pcb* duplication may have led to sub- or neo-functionalization given that *g6pcb1* and *g6pcb2* orthologs displayed opposite expression profiles in liver (Marandel et al., 2015). This hypothesis appears feasible as the mRNA levels of the other two gluconeogenic genes, *fbp1b1* and *pck2*, showed lower expression in land-locked salmon than in farmed salmon, suggesting reduced capacity for synthesis of glucose which, in this case, supports the glycaemia data. Thus, in contrast to the results obtained in land-locked salmon in the present study, inhibition of hepatic gluconeogenesis had not been previously observed in carnivorous fish fed high carbohydrate (Panserat et al., 2009; Kamalam et al., 2017). It must be noted that none of the actors of glucose metabolism involved in gluconeogenesis were detected in the present transcriptomic analysis, perhaps due to the absence of possibilities of distinction between the different orthologs.

### Lipid Metabolism in Liver: Diets and Populations Effects

Plasma triglyceride levels were affected by diet, with higher levels in carbohydrate-fed fish, which is not surprising given that lipogenesis plays an important role in carbohydrate metabolism in salmonids, with excess glucose transformed into fatty acids through *de novo* lipogenesis (Polakof et al., 2011a). This hypothesis is supported by the elevated expression of the lipogenic genes, *fas* and *g6pd*, in carbohydrate-fed salmon, which is consistent with results from a previous study (Jin et al., 2014). In agreement, transcriptomic data also showed that genes involved in lipid anabolism were highly represented and that the regulation was stronger when comparing fish from both populations fed diet CHO (FCHO vs. GCHO) denoting the strong relation between carbohydrate intake and lipid biosynthesis.

Plasma triglyceride levels were also affected by population with land-locked salmon showing much higher levels than farmed fish. However, these high levels in land-locked salmon were not accompanied by higher mRNA levels of the lipogenic enzymes *acly* or *fas* in liver, which does not explain why plasma triglyceride levels were higher in this population. This could be due to the fact that, as previously stated, land-locked salmon appeared to show a faster response in the regulation of glucose levels and, therefore, to convert carbohydrate into lipid via *de novo* synthesis, which in turn explains higher triglyceride levels in plasma. Consistent with these results, according to both transcriptomic and qPCR data, there was increased expression of catabolic genes such as *cpt1b* and *phosphatidylserine decarboxylase* in land-locked salmon that may be a mechanism to control plasma hyperlipidemia. In this respect, up-regulation of *cpt1* is a common finding when teleosts are fed high lipid or n-3 LC-PUFA (Peng et al., 2014; Xiao et al., 2017). In contrast, inhibition of expression of lipolysis genes (including *cpt2*) was detected in land-locked salmon in comparison with farmed fish (Betancor et al., 2016), which may indicate a more limited utilization of lipid as a source of energy in land-locked salmon. However, in the previous studies, feeds devoid of carbohydrate were used and, given the strong relationship between glucose and lipid utilization in animals, it is difficult to draw a firm conclusion. However, it must also be noted that the protein content of the feeds also varied (65% in NoCHO vs. 42% in CHO, but was always higher that the protein requirement for salmon) in order to balance the energy contents of the feeds and thus the effect/interaction between glucose and protein could explain differences to previous trials. However, another difference compared to the previous trial is that the present trial was performed in seawater with post-smolts, which may pose a stress for land-locked strain. However, this was essential as, for a trait of land-locked fish to be commercially valuable, it would have to have be beneficial when fish are reared under normal farming conditions.

Surprisingly, among the genes strongly influenced by population, there was one represented by two features, *elongation of very long chain fatty acids protein 6* (*elovl6*), which has been previously identified as a pivotal player in energy metabolism and insulin sensitivity in mice (Matsuzaka and Shimano, 2009). ELOVL6 is better known for its role in catalyzing the elongation of saturated and monounsaturated fatty acids with 12, 14, and 16 carbons (Moon et al., 2001), however, in teleosts, its expression does not appear to be affected by dietary fatty acid profile, with hepatic expression of this enzyme being very low (Xue et al., 2014). It was shown that deficiency of this enzyme in mice leads to a down-regulation of lipogenesis together with increased insulin sensitivity (Matsuzaka and Shimano, 2009). Given that, in the present study, the expression of *elovl6* was considerably lower in land-locked salmon, particularly when the high carbohydrate feed was provided, it is feasible to suggest that lower activity of this enzyme could reflect enhanced use of dietary carbohydrate by this population. In addition, land-locked salmon also showed lower expression of *acly*, and *g6pd* genes involved in the biosynthesis of pentose phosphate. Therefore, these data appear to reinforce the hypothesis that land-locked salmon are capable of consuming and metabolizing dietary starch for energy, either directly or by storage as lipid.

### Mitochondrial Metabolism in Liver: Diets and Populations Effects

Apart from being stored as glycogen or lipid, the fate of hydrolysed glucose can be to enter the Krebs (citrate) cycle and to produce ATP within mitochondria. Indeed the consumption of high-carbohydrate feed led to increased expression of the hepatic genes, *qcr2* and *cs*, involved in energy metabolism in mitochondria. These data may reflect higher activity toward the formation of ATP in fish fed high levels of dietary carbohydrate regardless of population. However, population seemed to have a greater influence than diet on energy metabolism as all the genes evaluated were lower in liver of land-locked salmon irrespective of dietary carbohydrate intake. Similarly, reduced expression of most of these genes occurred in sea bream fasted for 10 days (Bermejo-Nogales et al., 2015). In agreement with the candidate qPCR gene expression data, transcriptomic analysis of the liver indicated that, among the pathways altered by the genetic background of the salmon, there was a high representation (8 genes) of “citrate cycle” genes including *sdha*, which may indicate different regulation of the Krebs cycle in land-locked salmon irrespective of dietary content of carbohydrate. This decreased capacity of mitochondrial metabolism in liver of land-locked salmon is clearly a key signature of this salmon population (as confirmed by data in muscle, data not shown).

## Conclusion

Although we cannot exclude an effect of dietary protein level on the regulation of hepatic metabolism, the presence (at high level) and the absence of dietary digestible carbohydrate (starch) was the major difference between the two diets which could impact hepatic metabolism. Indeed, the results of the present study demonstrated that there were some clear differences between the two strains in plasma metabolites (glucose and triglyceride levels) as well as gene expression of glucose metabolism and mitochondrial actors 8 h after feeding high carbohydrate (starch) feed. All these data are promising and suggest different regulatory capacities between land-locked salmon and the farmed stock. However, further analysis are necessary in order to fully characterize the capacity of landlocked salmon to utilize dietary carbohydrate through long term experiments (at least 12 weeks of feeding the experimental feeds) and/or a glucose tolerance test. The pattern of hepatic gene expression after high dietary CHO intake was similar in land-locked and farmed salmon, generally lower FC were observed in land-locked fish reflecting the more rapid return to homeostasis, probably reflecting their natural adaptation to dietary CHO. Liver transcriptome analysis confirmed the results obtained by qPCR highlighting some new candidate genes such as *elovl6* to evaluate in studies assessing the capacity of salmonids to cope with high dietary carbohydrate. Land-locked salmon has again proved to be a potentially highly valuable genetic resource.

## Author Contributions

MB performed the transcriptomic analysis and drafted the manuscript. LM performed the qPCR analysis. OS and AM participated in fish husbandry and sampling. RO, DT, and SP designed the study and edited the manuscript.

## Conflict of Interest Statement

The authors declare that the research was conducted in the absence of any commercial or financial relationships that could be construed as a potential conflict of interest.
