# Supplementary material for: Impact of Dietary Carbohydrate/Protein Ratio on Hepatic Metabolism in Land-Locked Atlantic Salmon (Salmo salar L.)
Source: Front Physiol. 2018 Dec 6;9:1751. doi: 10.3389/fphys.2018.01751 (PMC6291493; doi:10.3389/fphys.2018.01751)
Supplement: Supplementary file 1 [file Table_1.DOCX]

**Dietary carbohydrates in land-locked Atlantic salmon (*Salmo salar* L.): impact on hepatic metabolism**

Betancor MB^1*^, Olsen RE^2^, Marandel L^3^, Skulstad, OF^4^, Madaro, A^4^, Tocher DR^1^, Panserat S^3^

^1^Institute of Aquaculture, Faculty of Natural Sciences, University of Stirling, Stirling FK9 4LA, United Kingdom

^2^Norwegian University of Science and Technology, Department of Biology, 7491 Trondheim, Norway

^3^INRA-UPPA, UMR 1419 Nutrition Metabolism Aquaculture, Institut National de la Recherche Agronomique, Aquapôle, Saint-Pée-sur-Nivelle, France

^4^Institute of Marine Research, 5984 Matredal, Norway

^*^ Correspondence: Mónica B Betancor; [m.b.betancor@stir.ac.uk](mailto:m.b.betancor@stir.ac.uk)

| **Genes** | **Proteins** | **Forward primers 5’-3’** | **Reverse primers 5’-3’** |
| --- | --- | --- | --- |
| ef1 | Elongation factor 1-α | TCCTCTTGGTCGTTTCGCTG | ACCCGAGGGACATCCTGTG |
| glut2b | Glucose transporter type 2 | CTATCAGAGAACGGTACAGGG | CAGGAAGGATGACACCACG |
| gckb | Glucokinase | TCTGTGCTAGAGACAGCCC | CATTTTGACGCTGGACTCCT |
| pkl | Pyruvate kinase | CCATCGTCGCGGTAACAAGA | GCCCCTGGCCTTTCCTATGT |
| g6pca | Glucose-6-phosphatase | GATGGCTTGACGTTCTCCT | AGATCCAGGAGAGTCCTCC |
| g6pcb1 | Glucose-6-phosphatase | AGGGACAGTTCGAAAATGGAG | CCAGAGAGGGAAGAAGATGAAGA |
| fbp1a | Fructose-1,6-biphospatase | GACAGAGGACGACCCGTG | GTACTGACCGGGTCCAACAT |
| fbp1b1 | Fructose-1,6-biphosphatase | CTCTCAAGAACCTCTACAGCCT | TCAGTTCTCCCGTTCCCTTC |
| fbp1b2 | Fructose-1,6-biphosphatase | ATCAGCAGGAATAGGTCGCG | CCTCCTCCAGCACGAATCTC |
| pck2 | Phosphoenolpyruvate carboxykinase type 2 | ACAATGAGATGATGTGACTGCA | TGCTCCATCACCTACAACCT |
| fas | Fatty acid synthase | TGATCTGAAGGCCCGTGTCA | GGGTGACGTTGCCGTGGTAT |
| acly | ATP citrate synthase | GCTTTTGCCACGGTGGTCTC | GCTTCCGCTACGCCAATGTC |
| g6pd | Glucose-6-phosphate dehydrogenase | CTCATGGTCCTCAGGTTTG | AGAGAGCATCTGGAGCAAGT |
| qcr2 | Ubiquinol cytochrome C reductase core protein 2 | CGTCACGCCTAGACTTCCTC | CTTCCAGGATCGGTGTGTTT |
| atp5a | ATP synthase | AGGTGGCTGGTACCATGAAG | TCTTGGAAGGGTCCATCTTG |
| cox4 | Cytochrome C Oxydase subunit 4 | TACGTGGGGGACATGGTGTT | CCCAGGAGCCCTTCTCCTTC |
| sdhb | Succinate dehydrogenase iron sulfur subunit | CCCAGGATCAAGAAGTTCCA | TTAAGCAGGCCAGTGTGTTG |
| cs | Citrate synthase | GGCCAAGTACTGGGAGTTCA | CTCATGGTCACTGTGGATGG |
| cpt1a | Carnitine palmitoyl transferase 1 | TCGATTTTCAAGGGTCTTCG | CACAACGATCAGCAAACTGG |
| cpt1b | Carnitine palmitoyl transferase 1 | CCCTAAGCAAAAAGGGTCTTCA | CATGATGTCACTCCCGACAG |
| hoad | 3-hydroxyacylCoA-dehydrogenase | GGACAAAGTGGCACCAGCAC | GGGACGGGGTTGAAGAAGTG |

**Supplementary Table 1.-** List of primers used for qRT-PCR

**Supplementary Table 2**. Transcripts corresponding to the top 100 most significant features exhibiting common differential expression in the two contrasts *(FCHO vs GCHO and FNoCHO vs GNoCHO)*

|  |  |  |  | FCHOvsGCHO | | FNoCHOvsGNoCHO | |
| --- | --- | --- | --- | --- | --- | --- | --- |
|  | KEGG | Gene name | Probe | p value | FC | p value | FC |
| Metabolism (51.1%) | K01079 | Phosphoserine phosphatase | Ssa#STIR17042 | 0.000 | 14.49 | 0.003 | 6.19 |
| Energy | K01079 | Phosphoserine phosphatase | Ssa#STIR04348 | 0.000 | 4.64 | 0.000 | 3.50 |
|  | K02685 | DNA primase large subunit | Ssa#S32005649 | 0.024 | 4.50 | 0.018 | 2.65 |
| Carbohydrate | K00710 | Polypeptide N-acetylgalactosaminyltransferase | Omy#S34421972 | 0.006 | 33.71 | 0.017 | 8.39 |
|  | K11262 | Acetyl-CoA carboxylase / biotin carboxylase 1 | Ssa#STIR15782 | 0.000 | 17.60 | 0.029 | 2.76 |
|  | K02377 | GDP-L-fucose synthase | Ssa#S18863091 | 0.000 | 9.38 | 0.031 | 5.17 |
|  | K00140 | Malonate-semialdehyde dehydrogenase | Ssa#TC72386 | 0.009 | 8.23 | 0.039 | 2.28 |
|  | K00134 | Glyceraldehyde 3-phosphate dehydrogenase | SsaHomCont2_090 | 0.010 | -6.56 | 0.003 | -7.60 |
|  | K00011 | Aldehyde reductase | Ssa#STIR00108_4 | 0.000 | 5.55 | 0.042 | 3.07 |
|  | K01640 | Hydroxymethylglutaryl-CoA lyase | Ssa#DY695952 | 0.000 | 5.17 | 0.022 | 2.99 |
|  | K01230 | Mannosyl-oligosaccharide alpha-1,2-mannosidase | Omy#TC150602 | 0.020 | -5.09 | 0.016 | -1.72 |
|  | K00134 | Glyceraldehyde 3-phosphate dehydrogenase | Ssa#S31963713 | 0.009 | -4.48 | 0.000 | -4.49 |
| Lipid | K15013 | Long-chain-fatty-acid-CoA ligase | Ssa#S35532713 | 0.000 | 25.50 | 0.000 | 12.15 |
|  | K00665 | Fatty acid synthase | Omy#S18148126 | 0.000 | 15.09 | 0.016 | 2.53 |
|  | K14457 | 2-acylglycerol O-acyltransferase 2 | Ssa#S31963159 | 0.000 | 13.42 | 0.017 | 5.29 |
|  | K07431 | Sterol 12-alpha-hydroxylase | Ssa#DW582478 | 0.002 | 11.78 | 0.007 | 3.11 |
|  | K10203 | Elongation of very long chain fatty acids protein 6 | Ssa#DW178452 | 0.000 | 11.50 | 0.009 | 3.01 |
|  | K10203 | Elongation of very long chain fatty acids protein 6 | Ssa#STIR40430 | 0.000 | 10.97 | 0.001 | 4.33 |
|  | K08765 | Carnitine O-palmitoyltransferase 1, liver isoform | Ssa#S35514366 | 0.000 | -6.91 | 0.018 | -2.48 |
|  | K01613 | Phosphatidylserine decarboxylase | Omy#S15318995 | 0.000 | -5.69 | 0.003 | -5.04 |
|  | K00071 | Corticosteroid 11-beta-dehydrogenase isozyme 2 | Ssa#STIR19502 | 0.000 | -5.21 | 0.002 | -5.15 |
|  | K12350 | Sphingomyelin phosphodiesterase | Ssa#S23659857 | 0.001 | 4.84 | 0.023 | 3.86 |
| Aminoacid | K00544 | Betaine-homocysteine S-methyltransferase | Ssa#STIR15320 | 0.000 | 6.87 | 0.028 | 2.77 |
|  | K00544 | Betaine-homocysteine S-methyltransferase | Ssa#S23869428 | 0.001 | 5.26 | 0.016 | 2.95 |
| Translation (2.1%) | K02934 | Large subunit ribosomal protein L6e | Ssa#S18885712 | 0.022 | -5.82 | 0.004 | -8.17 |
| Transcription (2.1%) | K12854 | Pre-mRNA-splicing helicase BRR2 | Ssa#STIR08493 | 0.000 | 5.11 | 0.000 | 4.38 |
| Signalling (21.3%) | K06626 | Cyclin E | Ssa#STIR06110 | 0.049 | 7.09 | 0.020 | 7.68 |
|  | K05143 | Tumor necrosis factor receptor superfamily member 6B | Ssa#S35538084 | 0.006 | -6.44 | 0.020 | -2.93 |
|  | K09782 | Amphiregulin | Ssa#STIR12482 | 0.001 | -6.26 | 0.003 | -3.71 |
|  | K05853 | Ca2+ transporting ATPase, sarcoplasmic/endoplasmic reticulum | Ssa#TC101205 | 0.000 | 5.74 | 0.001 | 2.65 |
|  | K04666 | Nodal | Ssa#TC98500 | 0.002 | -5.60 | 0.047 | -3.36 |
|  | K06856 | Immunoglobulin heavy chain | Omy#gi58201861 | 0.006 | -5.18 | 0.000 | -5.91 |
|  | K05856 | Lymphocyte cell-specific protein tyrosine kinase | Ssa#S30270592_S | 0.022 | -4.98 | 0.001 | -8.42 |
|  | K04959 | Inositol 1,4,5-triphosphate receptor type 2 | Ssa#STIR15847 | 0.025 | -4.93 | 0.024 | -5.01 |
|  | K06793 | Versican core protein | Ssa#TC102149 | 0.009 | 4.56 | 0.039 | 3.67 |
|  | K05853 | Ca2+ transporting ATPase, sarcoplasmic/endoplasmic reticulum | Ssa#DW557275 | 0.000 | 4.52 | 0.002 | 5.80 |
| Folding (2.1%) | K13251 | Translocon-associated protein subunit gamma | Ssa#CL266Ctg1 | 0.002 | -9.38 | 0.008 | -1.66 |
| Transport (6.4%) | K06560 | Mannose receptor, C type | Ssa#S35577817 | 0.005 | -6.58 | 0.000 | -13.89 |
|  | K05031 | Cystic fibrosis transmembrane conductance regulator | Ssa#S18892351 | 0.000 | 6.54 | 0.036 | 2.89 |
|  | K06560 | Mannose receptor, C type | Ssa#STIR44600 | 0.012 | -4.62 | 0.001 | -6.14 |
| Replication (6.4%) | K02542 | DNA replication licensing factor MCM6 | Omy#CA385715 | 0.006 | 7.05 | 0.026 | 5.18 |
|  | K02541 | DNA replication licensing factor MCM3 | Ssa#S35699881 | 0.002 | 6.54 | 0.005 | 5.02 |
|  | K02540 | DNA replication licensing factor MCM2 | Ssa#TC82076 | 0.017 | 4.50 | 0.012 | 3.22 |
| Immune system (6.4%) | K03905 | Fibrinogen gamma chain | Ssa#S18835908 | 0.000 | 9.62 | 0.006 | 8.88 |
|  | K07363 | IL2-inducible T-cell kinase | Ssa#S32002167 | 0.005 | -5.12 | 0.009 | -5.34 |
|  | K03905 | Fibrinogen gamma chain | Omy#BE669053 | 0.000 | 4.83 | 0.002 | 6.82 |
| Miscellaneous (2.1%) | K09030 | Fos-like antigen 2 | Ssa#STIR22687 | 0.004 | -5.01 | 0.011 | -2.04 |

**Supplementary Table 3**.- Transcripts found to be differentially regulated both by microarray and quantitative PCR after comparing either farmed (F) or landlocked (G) salmon fed a diet containing high levels of carbohydrates (CHO).

|  | **FCHO vs GCHO** | | | | | |
| --- | --- | --- | --- | --- | --- | --- |
|  | Microarray | | | qPCR | | |
|  |  | FC | p |  | FC | p |
| *g6pcb1* | down | 1.4 | * | down | 1.3 | *** |
| *g6pd* | up | 3.2 | *** | up | 3.6 | *** |
| *acly* | up | 4.2 | *** | up | 1.3 | *** |
| *qcr2* | up | 1.3 | * | up | 1.6 | *** |
| *atp5a* | up | 1.7 | * | up | 1.8 | *** |
| *cox4* | up | 1.8 | *** | up | 1.7 | ** |
| *sdhb* | up | 1.6 | ** | up | 1.3 | * |

*g6pcb1*, glucose 6-phosphatase paralog b1; *g6pd*, glucose 6-phosphate dehydrogenase; *acly*, ATP citrate lyase; *qcr2*, ubiquitinol cytochrome c reductase core protein 2; *atp5a*, ATP synthase form 5; *cox4*, cytochrome oxidase 4; *sdhb*, succinate dehydrogenase complex iron sulfur subunit B.
